# *Vital Signs:* Trends in State Suicide Rates — United States, 1999–2016 and Circumstances Contributing to Suicide — 27 States, 2015

**DOI:** 10.15585/mmwr.mm6722a1

**Published:** 2018-06-08

**Authors:** Deborah M. Stone, Thomas R. Simon, Katherine A. Fowler, Scott R. Kegler, Keming Yuan, Kristin M. Holland, Asha Z. Ivey-Stephenson, Alex E. Crosby

**Affiliations:** ^1^Division of Violence Prevention, National Center for Injury Prevention and Control, CDC; ^2^Division of Analysis, Research, and Practice Integration, National Center for Injury Prevention and Control, CDC.

## Abstract

**Introduction:**

Suicide rates in the United States have risen nearly 30% since 1999, and mental health conditions are one of several factors contributing to suicide. Examining state-level trends in suicide and the multiple circumstances contributing to it can inform comprehensive state suicide prevention planning.

**Methods:**

Trends in age-adjusted suicide rates among persons aged ≥10 years, by state and sex, across six consecutive 3-year periods (1999–2016), were assessed using data from the National Vital Statistics System for 50 states and the District of Columbia. Data from the National Violent Death Reporting System, covering 27 states in 2015, were used to examine contributing circumstances among decedents with and without known mental health conditions.

**Results:**

During 1999–2016, suicide rates increased significantly in 44 states, with 25 states experiencing increases >30%. Rates increased significantly among males and females in 34 and 43 states, respectively. Fifty-four percent of decedents in 27 states in 2015 did not have a known mental health condition. Among decedents with available information, several circumstances were significantly more likely among those without known mental health conditions than among those with mental health conditions, including relationship problems/loss (45.1% versus 39.6%), life stressors (50.5% versus 47.2%), and recent/impending crises (32.9% versus 26.0%), but these circumstances were common across groups.

**Conclusions:**

Suicide rates increased significantly across most states during 1999–2016. Various circumstances contributed to suicides among persons with and without known mental health conditions.

**Implications for Public Health Practice:**

States can use a comprehensive evidence-based public health approach to prevent suicide risk before it occurs, identify and support persons at risk, prevent reattempts, and help friends and family members in the aftermath of a suicide.

## Introduction

In 2016, nearly 45,000 suicides (15.6/100,000 population [age-adjusted]) occurred in the United States among persons aged ≥10 years (*1*). From 1999 to 2015, suicide rates increased among both sexes, all racial/ethnic groups, and all urbanization levels (*2*,*3*). Suicide rates have also increased among persons in all age groups <75 years, with adults aged 45–64 having the largest absolute rate increase (from 13.2 per 100,000 persons [1999] to 19.2 per 100,000 [2016]) and the greatest number of suicides (232,108) during the same period (*1*,*3*). Suicide is the tenth leading cause of death and is one of just three leading causes that are increasing (*1*,*4*). In addition, rates of emergency department visits for nonfatal self-harm, a main risk factor for suicide, increased 42% from 2001 to 2016 (*1*). Together, suicides and self-harm injuries cost the nation approximately $70 billion per year in direct medical and work loss costs (*1*).

The National Strategy for Suicide Prevention (*5*) calls for a public health approach to suicide prevention with efforts spanning multiple levels (individual, family/relationship, community, and societal). Such a comprehensive approach underscores that suicide is rarely caused by any single factor, but rather, is determined by multiple factors. Despite this call to action, suicide prevention largely focuses on identifying and referring suicidal persons to mental health treatment and preventing reattempts (*6*). In addition to mental health conditions and prior suicide attempts, other contributing circumstances include social and economic problems, access to lethal means (e.g., substances, firearms) among persons at risk, and poor coping and problem-solving skills (*5*). Expanded awareness of these additional circumstances contributing to suicide risk and action to address them can help reach the national goal, established by the National Action Alliance of Suicide Prevention and the American Foundation for Suicide Prevention, of reducing the annual suicide rate 20% by 2025 (*7*). To assist states in achieving this goal, CDC analyzed state-specific trends in suicide rates and assessed the multiple contributing factors to suicide; this report presents options for strategies to include in comprehensive suicide prevention efforts that are based on the best available evidence.

## Methods

Suicide rates were analyzed for persons aged ≥10 years because determining suicidal intent in younger children can be difficult (*8*). Age-specific suicide counts were tabulated based on National Vital Statistics System coded death certificate records (*International Classification of Diseases, Tenth Revision,* underlying-cause-of death codes X60–X84, Y87.0, U03). Age-specific population estimates were obtained from U.S. Census Bureau/National Center for Health Statistics bridged-race population data releases.

National and state-level suicide rate estimates were calculated for six consecutive 3-year aggregate periods spanning 1999–2016 (1999–2001; 2002–2004; 2005–2007; 2008–2010; 2011–2013; and 2014–2016). Rate estimates were age-adjusted to the U.S. 2000 standard population and expressed per 100,000 persons per year. Age-adjusted suicide rate trends were modeled using the same 3-year data aggregates, employing weighted least-squares regression with inverse-variance weighting. Modeled rate trends are reported in terms of average annual percentage changes.

Characteristics of persons aged ≥10 years who died by suicide, with and without known mental health conditions, and the circumstances surrounding the suicides were compared in the 27 states* with complete data participating in CDC’s National Violent Death Reporting System (NVDRS) in 2015. NVDRS defines mental health conditions as disorders and syndromes listed in the *Diagnostic and Statistical Manual of Mental Disorders, Fifth Edition* (*9*), with the exception of problematic alcohol use and other substance use that are captured separately in NVDRS. NVDRS aggregates data from three primary data sources: death certificates, coroner/medical examiner reports (including toxicology), and law enforcement reports. A range of circumstances (relationship problems, life stressors, and recent or impending crises) have been identified as potential risk factors for suicide in NVDRS. Circumstances captured are those identified as contributing to suicide in coroner/medical examiner or law enforcement reports, which reflect information provided by family and friends at the time of death. Decedents could have experienced multiple circumstances. Decedents with and without known mental health conditions were compared using chi-square tests. Logistic regression analyses were used to estimate adjusted odds ratios (aORs) with 95% confidence intervals (CIs), controlling for sex, age group, and race/ethnicity.

## Results

The most recent overall suicide rates (representing 2014–2016) varied fourfold, from 6.9 (District of Columbia) to 29.2 (Montana) per 100,000 persons per year (Supplementary Table; https://stacks.cdc.gov/view/cdc/53785). Across the study period, rates increased in all states except Nevada (where the rate was consistently high throughout the study period), with absolute increases ranging from 0.8 per 100,000 (Delaware) to 8.1 (Wyoming). Percentage increases in rates ranged from 5.9% (Delaware) to 57.6% (North Dakota), with increases >30% observed in 25 states (Supplementary Table; https://stacks.cdc.gov/view/cdc/53785) (Figure).

**FIGURE:**
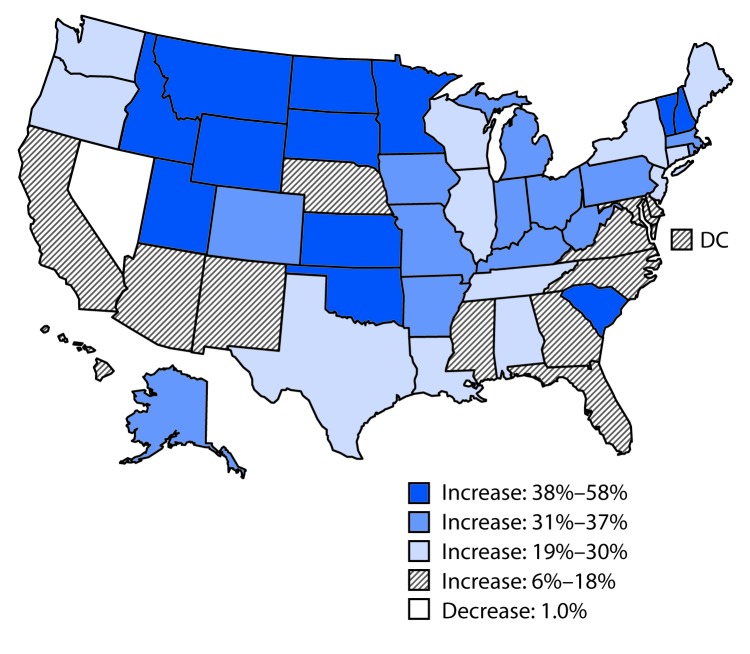
Percent change in annual suicide rate,* by state — United States, from 1999–2001 to 2014–2016 * Per 100,000 population, age-adjusted to the 2000 U.S. standard population.

Modeled suicide rate trends indicated significant increases in 44 states, among males (34 states) and females (43 states), as well as for the United States overall (Supplementary Table; https://stacks.cdc.gov/view/cdc/53785). Nationally, the model-estimated average annual percentage change for the overall suicide rate was an increase of 1.5%. By sex, estimated national rate trends further indicated significant average annual percentage change increases for males (1.1%) and females (2.6%) (Supplementary Table; https://stacks.cdc.gov/view/cdc/53785).

Suicide decedents without known mental health conditions (11,039; 54.0%) were compared with those with known mental health conditions (9,407; 46.0%) for 27 states. Whereas decedents were predominantly male (76.8%) (Table 1) and non-Hispanic white (83.6%), those without known mental health conditions, relative to those with mental health conditions, were more likely to be male (83.6% versus 68.8%; odds ratio [OR] = 2.3, 95% CI = 2.2–2.5) and belong to a racial/ethnic minority (OR range = 1.2–2.0). Suicide decedents without known mental health conditions also had significantly higher odds of perpetrating homicide followed by suicide (aOR = 2.9, 95% CI = 2.2–3.8). Among decedents aged ≥18 years, 20.1% of those without known mental health conditions and 15.3% of those with mental health conditions had previously served in the U.S. military or were serving at the time of death.

**TABLE 1:** Selected demographic and descriptive characteristics of suicides among persons aged ≥10 years with and without known mental health conditions — National Violent Death Reporting System, 27 states,* 2015

Characteristic	Total (N = 20,446) No. (%)	Known mental health condition^†^ (n = 9,407) No. (%)	No known mental health condition (n = 11,039) No. (%)	Chi-square p-value	OR^§^ (95% CI)	Adjusted OR^¶^ (95% CI)
**Sex**						
Male	**15,702 (76.8)**	6,469 (68.8)	9,233 (83.6)	<0.01	2.3 (2.2–2.5)	NA
Female	**4,744 (23.2)**	2,938 (31.2)	1,806 (16.4)	<0.01	0.4 (0.4–0.5)	NA
Age group (yrs)**						
10–24	**2,804 (13.7)**	1,211 (12.9)	1,593 (14.4)	<0.01	1.1 (1.1–1.2)	NA
25–44	**6,456 (31.6)**	3,036 (32.3)	3,420 (31.0)	<0.05	0.9 (0.9–1.0)	NA
45–64	**7,718 (37.7)**	3,820 (40.6)	3,898 (35.3)	<0.01	0.8 (0.8–0.8)	NA
≥65	**3,468 (17.0)**	1,340 (14.2)	2,128 (19.3)	<0.01	1.4 (1.3–1.5)	NA
**Race/Ethnicity**						
White, non-Hispanic	**17,102 (83.6)**	8,165 (86.8)	8,937 (81.0)	<0.01	0.6 (0.6–0.7)	NA
Black, non-Hispanic	**1,228 (6.0)**	411 (4.4)	817 (7.4)	<0.01	1.7 (1.5–2.0)	NA
American Indian/Alaska Native, non-Hispanic	**378 (1.8)**	112 (1.2)	266 (2.4)	<0.01	2.0 (1.6–2.6)	NA
Asian, non-Hispanic	**576 (2.8)**	235 (2.5)	341 (3.1)	<0.05	1.2 (1.1–1.5)	NA
Hispanic	**1,096 (5.4)**	463 (4.9)	633 (5.7)	<0.05	1.2 (1.0–1.3)	NA
Other	**66 (0.3)**	21 (0.2)	45 (0.4)	<0.05	1.8 (1.1–3.1)	NA
**Extended demographics**						
Ever served in military^††^	**3,429 (17.8)**	1,354 (15.3)	2,075 (20.1)	<0.01	1.4 (1.3–1.5)	1.1 (1.0–1.1)
Homeless	**240 (1.2)**	104 (1.1)	136 (1.3)	NS	1.1 (0.9–1.5)	1.2 (0.9–1.5)
**Incident type**						
Single suicide	**20,063 (98.2)**	9,318 (99.1)	10,745 (97.4)	<0.01	0.3 (0.3–0.4)	0.4 (0.3–0.5)
Homicide followed by suicide	**319 (1.6)**	64 (0.7)	255 (2.3)	<0.01	3.5 (2.6–4.5)	2.9 (2.2–3.8)
Multiple suicides	**64 (0.3)**	25 (0.3)	39 (0.4)	NS	1.3 (0.8–2.2)	1.6 (0.9–2.6)
**Method**						
Firearm	**9,909 (48.5)**	3,821 (40.6)	6,088 (55.3)	<0.01	1.8 (1.7–1.9)	1.6 (1.5–1.7)
Hanging/Strangulation/Suffocation	**5,907 (28.9)**	2,940 (31.3)	2,967 (26.9)	<0.01	0.8 (0.8–0.9)	0.8 (0.7–0.8)
Poisoning	**3,003 (14.7)**	1,861 (19.8)	1,142 (10.4)	<0.01	0.5 (0.4–0.5)	0.6 (0.6–0.7)
Substance class causing death^§§^						
Other (e.g., over-the-counter)	**1,021 (34.0)**	666 (35.8)	355 (31.1)	<0.01	0.8 (0.7–0.9)	0.9 (0.7–1.0)
Opioids	**944 (31.4)**	608 (32.7)	336 (29.4)	NS	0.9 (0.7–1.0)	0.9 (0.8–1.1)
Antidepressants	**800 (26.6)**	644 (34.6)	156 (13.7)	<0.01	0.3 (0.2–0.4)	0.3 (0.3–0.4)
Benzodiazepines	**624 (20.8)**	468 (25.1)	156 (13.7)	<0.01	0.5 (0.4–0.6)	0.5 (0.4–0.6)
Antipsychotics	**219 (7.3)**	195 (10.5)	24 (2.1)	<0.01	0.2 (0.1–0.3)	0.2 (0.1–0.3)
Other	**1,595 (7.8)**	780 (8.3)	815 (7.4)	<0.05	0.9 (0.8–1.0)	0.9 (0.8–1.0)
**Toxicology results**						
Any toxicology testing	**13,317 (65.1)**	6,658 (70.8)	6,659 (60.3)	<0.01	0.6 (0.6–0.7)	0.7 (0.6–0.7)
Positive for ≥1 substance^^¶¶^^	**9,913 (74.4)**	5,192 (78.0)	4,721 (70.9)	<0.01	0.7 (0.6–0.7)	0.8 (0.7–0.8)
Substance detected***						
**Alcohol**						
Tested	**10,950 (53.6)**	5,409 (57.5)	5,541 (50.2)	<0.01	0.7 (0.7–0.8)	0.8 (0.7–0.8)
Positive	**4,442 (40.6)**	2,115 (39.1)	2,327 (42.0)	<0.01	1.1 (1.0–1.2)	1.2 (1.1–1.3)
**Opioids**						
Tested	**8,554 (41.8)**	4,258 (45.3)	4,296 (38.9)	<0.01	0.8 (0.7–0.8)	0.8 (0.8–0.9)
Positive	**2,279 (26.6)**	1,238 (29.1)	1,041 (24.2)	<0.01	0.8 (0.7–0.9)	0.9 (0.8–1.0)
**Benzodiazepines**						
Tested	**8,124 (39.7)**	4,226 (44.9)	3,898 (35.3)	<0.01	0.7 (0.6–0.7)	0.7 (0.7–0.8)
Positive	**2,464 (30.3)**	1,639 (38.8)	825 (21.2)	<0.01	0.4 (0.4–0.5)	0.5 (0.5–0.6)
**Cocaine**						
Tested	**7,978 (39.0)**	3,866 (41.1)	4,112 (37.2)	<0.01	0.9 (0.8–0.9)	0.9 (0.9–1.0)
Positive	**499 (6.3)**	216 (5.6)	283 (6.9)	<0.05	1.2 (1.0–1.5)	1.2 (1.0–1.5)
**Amphetamines**						
Tested	**7,615 (37.2)**	3,696 (39.3)	3,919 (35.5)	<0.01	0.9 (0.8–0.9)	0.9 (0.8–0.9)
Positive	**736 (9.7)**	376 (10.2)	360 (9.2)	NS	0.9 (0.8–1.0)	1.0 (0.8–1.1)
**Marijuana**						
Tested	**6,569 (32.1)**	3,127 (33.2)	3,442 (31.2)	<0.01	0.9 (0.9–1.0)	0.9 (0.9–1.0)
Positive	**1,471 (22.4)**	710 (22.7)	761 (22.1)	NS	1.0 (0.9–1.1)	0.9 (0.8–1.0)
**Antidepressants**						
Tested	**5,425 (26.5)**	3,103 (33.0)	2,322 (21.0)	<0.01	0.5 (0.5–0.6)	0.6 (0.6–0.7)
Positive	**2,214 (40.8)**	1,735 (55.9)	479 (20.6)	<0.01	0.2 (0.2–0.2)	0.2 (0.2–0.3)

**Abbreviations:** CI = confidence interval; NA = not adjusted; NS = not significant; OR = odds ratio.

* Alaska, Arizona, Colorado, Connecticut, Georgia, Hawaii, Kansas, Kentucky, Maine, Maryland, Massachusetts, Michigan, Minnesota, New Hampshire, New Jersey, New Mexico, New York, North Carolina, Ohio, Oklahoma, Oregon, Rhode Island, South Carolina, Utah, Vermont, Virginia, and Wisconsin.

^†^ Decedent had been identified as having a current diagnosis of mental health condition in coroner/medical examiner or law enforcement reports.

^§^ OR reflects the risk among those without known mental health condition relative to those with known mental health condition.

^¶^ Logistic regression was used to estimate adjusted OR with 95% CIs after controlling for sex, age group, and race/ethnicity. Known mental health condition was used as the reference group.

** Decedents were aged ≥10 years, as per standard in the suicide prevention literature. ^††^ Denominator is decedents aged ≥18 years with reported military service status.

^§§^ Denominator is decedents who died by poisoning, including overdose.

^¶¶^ Denominator is decedents with any toxicology testing.

*** Denominator for each positive group is the number tested for the substance in that group.

Whereas firearms were the most common method of suicide overall (48.5%), decedents without known mental health conditions were more likely to die by firearm (55.3%) and less likely to die by hanging/strangulation/suffocation (26.9%) or poisoning (10.4%) than were those with known mental health conditions (40.6%, 31.3%, and 19.8%, respectively). These differences remained significant in the adjusted models.

Toxicology testing was less likely to be performed for decedents without known mental health conditions. Among those with toxicology results, decedents without known mental health conditions were less likely to test positive for any substance overall (aOR = 0.8, 95% CI = 0.7–0.8), including opioids (aOR = 0.90, 95% CI = 0.81–0.99), but were more likely to test positive for alcohol (aOR = 1.2, 95%, CI = 1.1–1.3).

Information on circumstances surrounding suicide were available for all decedents with mental health conditions (9,407) and approximately 85% of those without known mental health conditions (9,357) in 27 states (Table 2). Persons without known mental health conditions were less likely to have any problematic substance use (aOR = 0.7, 95% CI = 0.7–0.8) than were persons with known mental health conditions. Whereas two thirds of decedents with known mental health conditions had a history of mental health or substance use treatment (67.2%), just over half (54.0%) were in treatment at the time of death.

**TABLE 2:** Circumstances preceding suicide among decedents aged ≥10 years with and without known mental health conditions — National Violent Death Reporting System, 27 states,* 2015

Characteristic	Total No. (%)	Known mental health condition^†^ No. (%)	No known mental health condition No. (%)	Chi-square p-value	OR^§^ (95% CI)	Adjusted OR^¶^ (95% CI)
**Suicide with known circumstances**	**18,764 (91.8)**	9,407 (100)	9,357 (84.8)	<0.01	N/A	N/A
**Mental health**						
Any current diagnosed mental health condition**						
Depression/Dysthymia	**—^††^**	7,076 (75.2)	N/A	N/A	N/A	N/A
Anxiety disorder	**—^††^**	1,579 (16.8)	N/A	N/A	N/A	N/A
Bipolar disorder	**—^††^**	1,431 (15.2)	N/A	N/A	N/A	N/A
Schizophrenia	**—^††^**	509 (5.4)	N/A	N/A	N/A	N/A
PTSD	**—^††^**	424 (4.5)	N/A	N/A	N/A	N/A
ADD/ADHD	**—^††^**	226 (2.4)	N/A	N/A	N/A	N/A
Not specified	**—^††^**	760 (8.1)	N/A	N/A	N/A	N/A
Current depressed mood^§§^	**7,038 (37.5)**	3,962 (42.1)	3,076 (32.9)	<0.01	0.7 (0.6–0.7)	0.7 (0.6–0.7)
**Problematic substance use**						
Any	**5,319 (28.3)**	2,976 (31.6)	2,343 (25.0)	<0.01	0.7 (0.7–0.8)	0.7 (0.7–0.8)
Alcohol	**3,268 (17.4)**	1,862 (19.8)	1,406 (15.0)	<0.01	0.7 (0.7–0.8)	0.7 (0.7–0.8)
Other	**3,084 (16.4)**	1,768 (18.8)	1,316 (14.1)	<0.01	0.7 (0.7–0.8)	0.7 (0.7–0.8)
**Treatment**						
Current mental health/substance use treatment	**5,141 (27.4)**	5,077 (54.0)	64 (0.7)	<0.01	0.01 (0.01–0.01)	0.01 (0.01–0.01)
Ever treated for mental health/substance disorder	**6,717 (35.8)**	6,323 (67.2)	394 (4.2)	<0.01	0.02 (0.02–0.02)	0.02 (0.02–0.03)
**Relationship problems/loss**						
Any relationship problem/loss	**7,948 (42.4)**	3,726 (39.6)	4,222 (45.1)	<0.01	1.3 (1.2–1.3)	1.3 (1.2–1.4)
Intimate partner problem	**5,098 (27.2)**	2,270 (24.1)	2,828 (30.2)	<0.01	1.4 (1.3–1.5)	1.4 (1.3–1.5)
Perpetrator of interpersonal violence in past month	**414 (2.2)**	131 (1.4)	283 (3.0)	<0.01	2.2 (1.8–2.7)	2.0 (1.6–2.4)
Victim of interpersonal violence in past month	**84 (0.4)**	53 (0.6)	31 (0.3)	<0.05	0.6 (0.4–0.9)	0.8 (0.5–1.2)
Family relationship problem	**1,671 (8.9)**	873 (9.3)	798 (8.5)	NS	0.9 (0.8–1.0)	1.0 (0.9–1.1)
Other relationship problem (nonintimate)	**403 (2.1)**	202 (2.1)	201 (2.1)	NS	1.0 (0.8–1.2)	1.1 (0.9–1.3)
Argument or conflict (not specified)	**2,914 (15.5)**	1,278 (13.6)	1,636 (17.5)	<0.01	1.3 (1.2–1.5)	1.4 (1.3–1.5)
Death of a loved one (any)	**1,497 (8.0)**	826 (8.8)	671 (7.2)	<0.01	0.8 (0.7–0.9)	0.9 (0.8–0.9)
Nonsuicide death	**1,181 (6.3)**	647 (6.9)	534 (5.7)	<0.01	0.8 (0.7–0.9)	0.9 (0.8–1.0)
Suicide of family or friend	**379 (2.0)**	217 (2.3)	162 (1.7)	<0.01	0.7 (0.6–0.9)	0.8 (0.7–1.0)
**Other life stressors**						
Any life stressor	**9,171 (48.9)**	4,442 (47.2)	4,729 (50.5)	<0.01	1.1 (1.1–1.2)	1.1 (1.0–1.2)
Recent criminal legal problem	**1,588 (8.5)**	586 (6.2)	1,002 (10.7)	<0.01	1.8 (1.6–2.0)	1.7 (1.5–1.9)
Other legal problem	**748 (4.0)**	378 (4.0)	370 (4.0)	NS	1.0 (0.8–1.1)	1.0 (0.9–1.2)
Physical health problem	**4,179 (22.3)**	2,012 (21.4)	2,167 (23.2)	<0.01	1.1 (1.0–1.2)	1.0 (1.0–1.1)
Job/Financial problem^¶¶^	**2,941 (16.2)**	1,530 (16.8)	1,411 (15.6)	<0.05	0.9 (0.8–1.0)	0.9 (0.8–1.0)
Eviction or loss of home	**722 (3.8)**	317 (3.4)	405 (4.3)	<0.01	1.3 (1.1–1.5)	1.4 (1.2–1.6)
School problem***	**162 (19.9)**	70 (17.8)	92 (21.9)	NS	1.3 (0.9–1.8)	1.3 (0.9–1.9)
Recent release from an institution^†††^	**1,412 (7.6)**	941 (10.2)	471 (5.1)	<0.01	0.5 (0.4–0.5)	0.5 (0.4–0.5)
Jail/Prison/Detention facility	**203 (14.4)**	82 (8.7)	121 (25.7)	<0.01	3.6 (2.7–4.9)	4.5 (3.2–6.4)
Hospital	**517 (36.6)**	311 (33.0)	206 (43.7)	<0.01	1.6 (1.3–2.0)	1.3 (1.0–1.7)
Psychiatric hospital/institution	**469 (33.2)**	439 (46.7)	30 (6.4)	<0.01	0.1 (0.1–0.1)	0.1 (0.1–0.1)
Other (includes alcohol/SU treatment facilities)	**223 (15.8)**	109 (11.6)	114 (24.2)	<0.01	2.4 (1.8–3.3)	2.5 (1.8–3.3)
**Crisis within past or upcoming 2 weeks^^§§§^^**	**5,525 (29.4)**	2,444 (26.0)	3,081 (32.9)	<0.01	1.4 (1.3–1.5)	1.4 (1.3–1.5)
Intimate partner problem	**1,968 (35.6)**	854 (34.9)	1,114 (36.2)	NS	1.1 (0.9–1.2)	1.1 (0.9–1.2)
Physical health problem	**739 (13.4)**	315 (12.9)	424 (13.8)	NS	1.1 (0.9–1.3)	1.0 (0.8–1.2)
Criminal legal problem	**621 (11.2)**	203 (8.3)	418 (13.6)	<0.01	1.7 (1.5–2.1)	1.6 (1.3–1.9)
Family relationship problem	**430 (7.8)**	212 (8.7)	218 (7.1)	<0.05	0.8 (0.7–1.0)	0.9 (0.7–1.1)
Job problem	**354 (6.4)**	191 (7.8)	163 (5.3)	<0.01	0.7 (0.5–0.8)	0.7 (0.5–0.8)
**Suicide event/history**						
Left a note	**6,468 (34.5)**	3,182 (33.8)	3,286 (35.1)	NS	1.1 (1.0–1.1)	1.2 (1.1–1.2)
Disclosed suicide intent	**4,405 (23.5)**	2,306 (24.5)	2,099 (22.4)	<0.01	0.9 (0.8–1.0)	0.9 (0.8–0.9)
History of ideation	**5,990 (31.9)**	3,838 (40.8)	2,152 (23.0)	<0.01	0.4 (0.4–0.5)	0.4 (0.4–0.5)
History of attempts	**3,732 (19.9)**	2,770 (29.4)	962 (10.3)	<0.01	0.3 (0.3–0.3)	0.3 (0.3–0.3)

**Abbreviations:** ADD/ADHD = attention deficit disorder/attention deficit hyperactivity disorder; CI = confidence interval; N/A = not applicable; NS = not significant; OR = odds ratio; PTSD = posttraumatic stress disorder; SU = substance use.

* Alaska, Arizona, Colorado, Connecticut, Georgia, Hawaii, Kansas, Kentucky, Maine, Maryland, Massachusetts, Michigan, Minnesota, New Hampshire, New Jersey, New Mexico, New York, North Carolina, Ohio, Oklahoma, Oregon, Rhode Island, South Carolina, Utah, Vermont, Virginia, and Wisconsin.

^†^ Decedent had been identified as having a current diagnosis of mental health condition in coroner/medical examiner or law enforcement reports.

^§^ OR reflects the risk among those without known mental health condition relative to those with known mental health condition.

^¶^ Logistic regression was used to estimate adjusted OR with 95% CIs after controlling for sex, age group, and race/ethnicity. Known mental health condition was the reference group.

** Includes decedents with one or more diagnosed current mental health conditions, which are not mutually exclusive. Therefore, sums of percentages for the diagnosed conditions exceed 100%. Denominator includes the number of decedents with one or more current diagnosed mental health conditions.

^††^ The specific type of mental health condition was calculated only among those with one or more known diagnosed mental health conditions.

^§§^ Not a diagnosis.

^¶¶^ Denominator is decedents aged ≥18 years.

*** Denominator is decedents aged 10–18 years.

^†††^ Denominator of institution subgroup is decedents with recent release from an institution. Recent release from an institution is defined as having occurred within the past month.

^§§§^ Denominator of crisis subgroup is decedents with any crisis within past or upcoming 2 weeks. Crises depicted here represent the most commonly occurring categories.

Decedents without known mental health conditions had a significantly higher likelihood of any relationship problem/loss (45.1%) than did those with known mental health conditions (39.6%), specifically intimate partner problems (30.2% versus 24.1%), arguments/conflicts (17.5% versus 13.6%), and perpetrating interpersonal violence in the past month (3.0% versus 1.4%). Decedents without known mental health conditions were also more likely than were those with known mental health conditions to have experienced any life stressors (50.5% versus 47.2%) such as recent criminal legal problems (10.7% versus 6.2%) or eviction/loss of home (4.3% versus 3.4%) and were more likely to have had a recent or impending (within the preceding or upcoming 2 weeks, respectively) crisis (a current or acute event thought to contribute to the suicide) (32.9% versus 26.0%). All of these differences remained significant in the adjusted models. Physical health problems and job/financial problems were commonly contributing stressors among both persons without mental health conditions (23.2% and 15.6%, respectively) and those with mental health conditions (21.4% and 16.8%, respectively). Similarly, among all persons with recent crises, intimate partner problems were the most common types and did not differ by group.

Decedents without known mental health conditions had significantly lower odds of recent release from any institution (aOR = 0.5, 95% CI = 0.4–0.5). Among those recently released, decedents without known mental health conditions were significantly more likely than decedents with mental health conditions to have been released from a correctional facility (25.7% versus 8.7%), hospital (43.7% versus 33.0%), or other facility, such as an alcohol/substance use treatment facility (24.2% versus 11.6%). Among decedents with known mental health conditions who were recently released from an institution, 46.7% were released from psychiatric facilities.

Decedents without known mental health conditions were significantly less likely to have a history of suicidal ideation (23.0%) or prior suicide attempts (10.3%) compared with those with known mental health conditions (40.8% and 29.4%, respectively). Suicide intent was disclosed by 22.4% and 24.5% of persons without and with known mental health conditions, respectively.

## Conclusions and Comments

During 1999–2016, suicide rates increased significantly in 44 states, and 25 states experienced increases >30%. Rates increased significantly among males in 34 states, and females in 43 states. Additional research into the specific causes of these trends is needed. Data from the 27 states participating in NVDRS provide important insight into circumstances surrounding suicide and can help states identify prevention priorities.

Suicidologists regularly state that suicide is not caused by a single factor (*5*); however, suicide prevention is often oriented toward mental health conditions alone with regard to downstream identification of suicidal persons, treatment of mental health conditions, and prevention of reattempts. This study found that approximately half of suicide decedents in NVDRS did not have a known mental health condition, indicating that additional focus on nonmental health factors further upstream could provide important information for a public health approach (*10*). Those without a known mental health condition suffered more from relationship problems and other life stressors such as criminal/legal matters, eviction/loss of home, and recent or impending crises.

Similarly, persons with mental health conditions also often experienced other circumstances such as relationship problems and job/financial or physical health problems that contributed to their suicide. These findings point to the need to both prevent the circumstances associated with the onset of mental health conditions and support persons with known mental health conditions to decrease their risk for poor outcomes (*11*). Two thirds of suicide decedents with mental health conditions had a history of treatment for mental health or substance use disorders, with approximately half in treatment when they died. This finding suggests the need for additional safety supports, including broader implementation of affordable and effective treatment modalities, such as doctor-patient collaborative care models and proven cognitive-behavioral therapies. In addition, increased access to behavioral health providers in underserved areas is needed, as is expansion of health care systems that integrate physical and behavioral health, with a priority on suicide prevention and patient safety, especially through care transitions (*12*).

Comprehensive statewide suicide prevention activities are needed to address the full range of factors contributing to suicide. Prevention strategies include strengthening economic supports (e.g., housing stabilization policies, household financial support); teaching coping and problem-solving skills to manage everyday stressors and prevent future relationship problems, especially early in life; promoting social connectedness to increase a sense of belonging and access to informational, tangible, emotional, and social support; and identifying and better supporting persons at risk (e.g., military veterans, persons with physical/mental health conditions) (*12*). Other strategies include creating protective environments (e.g., reducing access to lethal means among persons at risk for suicide, creating organizational and workplace policies to promote help-seeking, easing transitions into and out of work for persons with mental health conditions and other life challenges), strengthening access to and delivery of care, supporting family and friends after a suicide, and encouraging the media to follow safe reporting recommendations (*12*). Some states, such as Colorado, are planning to implement such a comprehensive approach to suicide prevention (*10*).

The findings in this report are subject to at least three limitations. First, in the state-level analysis, rankings for four states (Maryland, Massachusetts, Rhode Island, and Utah) might have been affected by large proportions of injury deaths of undetermined intent (potentially biasing reported suicide rates downward) or decreased percentages of such deaths over time (potentially biasing estimated rate trends upward). Second, NVDRS is not yet nationally representative; the 27 states included represent 49.6% of the population (https://factfinder.census.gov/faces/tableservices/jsf/pages/productview.xhtml). Finally, abstractors of NVDRS data are limited to information contained in investigative reports. Therefore, the extent of informant knowledge can affect data completeness and accuracy. Studies that include more in-depth interviews with next-of-kin often identify greater attributions to mental health disorders (*13*); however, many methodological variations across studies exist (*14*). It is likely that some persons without known mental health conditions in the current study were experiencing mental health challenges that were unknown, undiagnosed, or not reported by key informants. Nonetheless, the high prevalence of diverse contributing circumstances among those with and without known mental health conditions suggests the importance of addressing the broad range of factors that contribute to suicide.

Suicide is a growing public health problem. Effective approaches to prevent the many suicide risk factors are available. States and communities can use data from NVDRS and resources such as CDC’s *Preventing Suicide: A Technical Package of Policy, Programs, and Practices* (*12*) to better understand suicide in their populations, prioritize evidence-based comprehensive suicide prevention, and save lives.

**Summary**

**What is already known about this topic?**

In 2016, nearly 45,000 persons died by suicide in the United States. Mental health conditions are one of several contributors to suicide.

**What is added by this report?**

During 1999–2016, suicide rates increased in nearly every state, including >30% increases in 25 states. 2015 data from 27 states indicate 54% of suicide decedents were not known to have mental health conditions. Relationship, substance use, health, and job or financial problems are among the other circumstances contributing to suicide.

**What are the implications for public health practice?**

A comprehensive approach using proven prevention strategies, such as those in CDC’s *Preventing Suicide: A Technical Package of Policy, Programs, and Practices*, can help reach the national goal of reducing the annual suicide rate 20% by 2025.

## Footnotes

* Alaska, Arizona, Colorado, Connecticut, Georgia, Hawaii, Kansas, Kentucky, Maine, Maryland, Massachusetts, Michigan, Minnesota, New Hampshire, New Jersey, New Mexico, New York, North Carolina, Ohio, Oklahoma, Oregon, Rhode Island, South Carolina, Utah, Vermont, Virginia, and Wisconsin.
